# Gastrointestinal Carriage of Vancomycin-Resistant Enterococci and Carbapenem-Resistant Gram-Negative Bacteria in an Endemic Setting: Prevalence, Risk Factors, and Outcomes

**DOI:** 10.3389/fpubh.2020.00055

**Published:** 2020-03-18

**Authors:** Alexandra Vasilakopoulou, Polyxeni Karakosta, Sophia Vourli, Aikaterini Tarpatzi, Paraskevi Varda, Maria Kostoula, Anastasia Antoniadou, Spyros Pournaras

**Affiliations:** ^1^Clinical Microbiology Laboratory, Medical School, Attikon University General Hospital, National and Kapodistrian University of Athens, Athens, Greece; ^2^Infection Control Committee, Attikon University General Hospital, Athens, Greece; ^3^4th Department of Internal Medicine, Medical School, Attikon University General Hospital, National and Kapodistrian University of Athens, Athens, Greece

**Keywords:** vancomycin-resistant enterococci, carbapenem-resistance, carriage, risk-factors, mortality, length of stay

## Abstract

**Background:** Gastrointestinal carriage of vancomycin-resistant enterococci (VRE) and carbapenem-resistant Gram-negative bacteria (CRGN) constitutes a major public health concern as it may be followed by clinical infection development or lead to intra-hospital dissemination. Detection of carriers and implementation of infection control measures are essential in every hospital. In this study we determined the point prevalence of VRE and CRGN in the fecal flora of the inpatients of a tertiary university hospital in Greece. We determined risk factors for carriage and examined the impact of carriage on hospital outcomes.

**Materials/Methods:** A point prevalence study of VRE/CRGN rectal carriage of inpatients was conducted on March 2018. Specimens were selectively cultured for VRE/CRGN, microorganisms were biochemically identified, submitted to antibiotic susceptibility testing, and tested for carbapenemase production. Data on potential risk factors and hospital outcomes were collected at the time of culture and until hospital discharge. Multivariable logistic and linear regression models were used, adjusting for confounders.

**Results:** Four hundred ninety-one patients were enrolled in the study. Of them, 64 (13.0%) were positive for VRE carriage, 40 (8.2%) for CRGN, and 10 patients (2.1%) for both VRE and CRGN. VRE carriage was independently associated with age over 65 years (adjusted OR: 2.4 [95%CI: 1.3, 4.5]) and length of stay (LOS) before rectal sampling (OR: 1.1 [95%CI: 1.0, 1.1]). Carriage of CRGN was associated with 11 days increase of LOS after rectal sampling (β-coef: 11.4 [95%CI: 1.6, 21.2]), with a 3.5-fold increased risk of acquiring a resistant pathogen after rectal swabbing (RR: 3.5 [95%CI 1.2, 9.9]) and with a 6-fold increased risk of mortality (RR: 6.1 [95%CI: 2.1, 17.9]), after adjusting for sex, age, and comorbidity index.

**Conclusions:** High prevalence rates were found for VRE and CRGN carriage among the inpatients of our hospital. Prolonged hospitalization and age were independent risk factors for VRE carriage, while CRGN carriage was associated with increased risk of acquiring a resistant pathogen, prolonged hospital stay, and increased mortality.

## Introduction

The wide dissemination of carbapenem-resistant Gram-negative bacteria (CRGN) and vancomycin-resistant enterococci (VRE) limits therapeutic alternatives and represents a global public health threat (1). The consequences of multidrug-resistant (MDR) bacterial infections include high morbidity and mortality and considerable economic loss (2). A recent study estimated the impact of infections caused by antimicrobial-resistant bacteria in countries within the EU and the European Economic Area for 2015. It was estimated that ~670,000 infections with resistant bacteria were documented in EARS-Net data, with these infections accounting for ~33,000 attributable deaths and 870,000 disability-adjusted life-years (DALYs). Notably, Greece and Italy contributed the highest burden among all participating countries and, for Greece, most of the infections were due to carbapenem- or colistin-resistant bacteria (3). The hospital environment seems to serve as the breeding grounds for MDR organisms (MDRO) (4). Asymptomatic rectal carriage of these organisms may precede infection and constitutes a reservoir for transmission that may remain unidentified in hospitals that do not implement active surveillance testing (5).

While Greece is considered one of the most common countries in Europe for antimicrobial resistance (6), only a limited number of studies have focused on MDRO rectal carriage and colonization to assess their prevalence, risk factors, and associated adverse outcomes (7–11). A previous study from our hospital has reported a prevalence of 14.3% of VRE carriage among hospitalized patients, identified invasive devices and duration of antimicrobial treatment as risk factors, and found that VRE carriage was not an independent predictor of mortality (12). Similar studies from other regions have reported VRE carriage rates ranging from 2 to 37% (13–15), while prevalence rates for CRGN rectal carriage ranged from 5.3 to 52% (16–18). At the same time, there is an increased risk of carbapenem-resistant enterobacteriaceae (CRE) infection and mortality in patients who test positive for carriage of CRE (19, 20). To the best of our knowledge, there is no previous study from Greece focusing on both VRE and CRGN rectal carriage, exploring respective risk factors and adverse outcomes.

The primary objective of the present study was: (i) to determine the prevalence of rectal carriage of VRE and CRGN, (ii) to identify risk factors for VRE/CRGN rectal carriage, and (iii) to examine the impact of VRE/CRGN rectal carriage on hospital outcomes in inpatients of a University General Hospital in Greece.

## Materials and Methods

### Subjects

The University Hospital “Attikon” in Athens is a modern tertiary care teaching hospital and is the largest in the West Attica region (2,000,000 population), with 750 beds in total and >71.000 admissions/year. The hospital attends to cases of high complexity in internal medicine and surgery.

The first part of the present project was a point prevalence study of VRE/CRGN rectal carriage of hospital patients that was conducted on March 22nd and 23rd, 2018. Adult patients hospitalized in all surgical and internal medicine departments were surveyed by obtaining rectal swab cultures. In total, 17 medical and surgical wards participated in the study: general internal medicine, cardiology, dermatology, neurology, respiratory medicine, obstetrics/gynecology, cardiothoracic surgery, neurosurgery, urology, otorhinolaryngology, orthopedics, vascular surgery, hematology, oncology, nephrology, gastroenterology, and general surgery. The special and intensive care units were not included in the study, as they were already on active surveillance for VRE/CRGN carriage. The psychiatric ward was also excluded because patients lacked the mental competency necessary to participate. The second part of the project was a cohort study that included all participants from the first part and followed them from the time of rectal swab culture until death or discharge from the hospital. Face-to-face completed questionnaires together with medical records and communication with physicians were used to obtain information on potential risk factors at the time of rectal swabbing, while all examined outcomes were extracted from medical records retrospectively. The study was approved by the institutional review board of the hospital (62,17/10/2017) and all patients provided informed consent after a complete description of the study.

### Culture, Identification, and Susceptibility Testing

A rectal swab was obtained from every consenting hospitalized patient. The swabs were transferred by using transport swabs in Amies transport medium (Biomedics, Madrid, Spain) and were transported to the microbiology laboratory for selective culture of VRE and CRGN. Bile-esculin agar with vancomycin (6 mg/L) and MacConkey agar with meropenem (1 mg/L) were used for selective cultivation. Microorganisms were biochemically identified by Phoenix automated microbiology system (BD Diagnostic Systems, Sparks, MD) and submitted to antibiotic susceptibility testing, according to EUCAST 2018 guidelines and breakpoints. The combination disk test was used for screening carbapenemase production using meropenem 10 μg disks (BIO-RAD, Marnes-la-Coquette, France) with or without inhibitors [phenyl boronic acid (PBA), ethylenediaminetetraacetic acid (EDTA)] (21). The guidelines of the European Committee on Antimicrobial Susceptibility Testing (EUCAST) were applied for detection of resistance mechanisms and specific resistances of clinical and/or epidemiological importance (22). A meropenem disk with PBA and EDTA was also included to detect double carbapenemase producers (KPC and VIM), which have been found in Greek hospitals since 2009 (21, 23). The immunochromatographic assay, NG-test CARBA 5 (NG Biotech, 35480 Guipry, France) that discriminates KPC, IMP, VIM, NDM, and OXA-48-like producers, was also used.

### Rectal Carriage of VRE/CRGN

All patients that tested positive for VRE and/or CRGN in the rectal swab culture were defined as carriers. Standard infection control measures to reduce transmission were used in these cases. The health personnel had to implement contact precautions (gloves, gowns) for all encounters with the carriers. The wards focused on thorough cleaning of the environment surrounding the positive patient, especially the patient care equipment. Whenever it was possible, the patient was isolated in a single room (24, 25).

### Risk Factors for VRE/CRGN Rectal Carriage

Following informed consent and after obtaining the rectal swab, every hospitalized patient completed a face-to-face questionnaire which was captured in a standardized form. These data, along with parameters retrieved from the patients' records, included information on age, gender, ward, length of stay (LOS) before rectal swabbing, transfer from another hospital, comorbidities, presence of indwelling medical devices, chronic immobilization, last year hospitalization or ICU admission, and specific therapies, such as immunosuppressive therapy, antineoplastic, or antimicrobial chemotherapy.

Comorbidities included: chronic kidney disease, diabetes, dermatologic lesions, hematological malignancy, solid organ malignancy, metastatic disease, neurologic disease, heart failure, coronary artery disease, chronic liver disease, cerebrovascular disease, peripheral vascular disease, and chronic obstructive pulmonary disease. A slightly modified Charlson comorbidity index (CCI) (26) that predicts the 10-years mortality for a patient having a range of 17 comorbid conditions was also calculated. Each condition is assigned with a score of 1, 2, 3, or 6, depending on the risk of death associated with this condition; the scores are then summed up and give a total score which predicts mortality. The clinical conditions and scores are as follows: One for each: myocardial infarct, heart failure, peripheral vascular diseases, dementia, cerebrovascular disease, chronic lung diseases, connective tissue diseases, ulcer, and mild chronic liver diseases. Two for each: hemiplegia, moderate or severe kidney diseases, diabetes with or without complications, tumor, leukemia, lymphoma. Three for each: moderate or severe liver disease. Six for each: metastatic solid tumor, AIDS. In our modified version of CCI (modified CCI), we used chronic immobilization instead of hemiplegia and we did not use data on mild chronic liver diseases, since they were not present in any patient.

Indwelling medical devices included central lines, urinary catheters, pacemakers, and other devices, such as external wound drains, enteral feeding tubes, endotracheal tubes, and tracheostomies.

Chronic immobilization is defined as loss of anatomical movement due to alteration of physiological function, which in daily practice is commonly defined as more than three-day-bed rest or inability to perform mobile activity on a bed, transfer, or ambulation (27, 28).

Last year, hospitalization or ICU admission included hospitalization in an acute care hospital (ward or ICU, respectively) for two or more days in the past 1 year.

Previous antibiotic therapy was defined as prescription of antibiotics for at least 2 days within the past 1 year. Immunosuppressive therapy included administration of steroids, cyclophosphamide, azathioprine, methotrexate, mycophenolate mofetil, and calcineurin inhibitors during the last year.

### Hospital Outcomes

Hospital outcomes were collected prospectively during hospitalization and included: (i) Mortality: death from any cause during hospitalization, (ii) Hospital LOS, (iii) LOS after rectal swabbing, and (iv) Isolation of a resistant pathogen [VRE, methicillin-resistant *Staphylococcus aureus* (MRSA), carbapenem-resistant Gram-negative bacteria and Gram-negative bacteria resistant to three or more of: beta-lactams, aminoglycosides, quinolones, co-trimoxazole]. from: (a) any clinical culture, and (b) blood, after rectal swabbing.

### Statistical Analysis

Statistical analysis was performed using the statistical package STATA, version 13 (StataCorp, College Station, TX). Univariate associations between background characteristics and VRE/CRGN rectal carriage were studied using Pearson's chi-square test for categorical variables (with Fisher's exact test for groups with <5 subjects expected in a cell) and non-parametric Kruskal–Wallis tests for continuous non-normally distributed variables (tested by the Shapiro–Wilk normality test). Since potential risk factors and VRE/CRGN rectal carriage were measured in a cross-sectional design, associations of potential risk factors with VRE/CRGN rectal carriage were estimated with univariate logistic regression models. Estimated associations were described as odds ratios (OR) with 95% confidence intervals. All variables with a *p* ≤ 0.050 in univariate analysis were included in a multiple regression model to examine independent risk factors for VRE/CRGN rectal carriage. In the prospective part of our study and in order to estimate the risk of VRE/CRGN rectal carriage on hospital outcomes, we used multivariable log-binomial or log-Poisson (if convergence failed) regression models to estimate relative risks (RRs) with 95% CIs for categorical outcomes (resistant pathogen in any culture and in blood after rectal swabbing, mortality) and multivariable linear regression models to estimate β coefficients with 95% CIs for continuous outcomes (hospital LOS, LOS after rectal swab). Modified Charlson Comorbidity Index, age, and sex were included as confounders *a priori* in all analyses. All association testing was conducted assuming a *p* ≤ 0.050 significance level.

## Results

**Figure 1:**
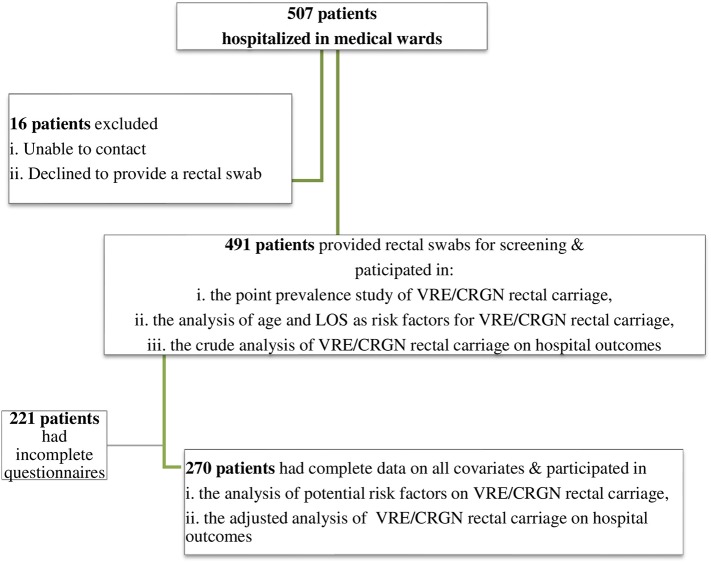
Flow diagram depicting the patients that participated in: (i) the point prevalence study of VRE/CRGN rectal carriage, (ii) the analysis of potential risk factors on VRE/CRGN rectal carriage, (iii) the analysis of VRE/CRGN rectal carriage on hospital outcomes.

In total, 507 adult patients hospitalized in medical wards were contacted to participate and 491 provided rectal swabs were included in the analysis (participation rate: 96.6%) (Figure 1). Baseline characteristics of the study population according to rectal carriage of CRGN and VRE are presented in Table 1. Forty patients were identified as CRGN carriers (prevalence: 8.2%) and 64 as VRE carriers (prevalence: 13.0%). Of the carrier patients, 30% were hospitalized in single rooms. All VRE isolates were identified as VanA-phenotype *E. faecium* (high level resistance to vancomycin and teicoplanin). Molecular detection of *van* genes was not performed The respective prevalence for carbapenem-resistant (CR) enterobacteriaceae (CRE), CR-*Acinetobacter spp.*, and CR-*Pseudomonas spp*. carriers was 5.9, 1.8, and 1.2%, respectively. Four different types of carbapenemases were detected in CRE colonized patients: KPC, 65.5% (*n* = 19); NDM, 24.1% (*n* = 7); VIM, 6.9% (*n* = 2); and OXA-48, 3.5% (*n* = 1). Patients colonized with VRE tended to be older (>65 years) compared to non-colonized patients. Regarding CRGN carriers, they were characterized by a higher modified CCI, were more likely to have a urine catheter, and to have been hospitalized in a medical ward/ICU or to have received antibiotics during the last year, compared with non-carriers. Moreover, both groups of CRGN and VRE carriers tended to have longer LOS before rectal swabbing. Mortality for non-carriers was 4.8% (*n* = 19), lower than that for CRGN (40%, *p* < 0.001) and VRE carriers (15.9%, *p* < 0.001). Hospital outcomes, such as hospital LOS and subsequent resistant pathogens isolated from blood, were also more frequent in both subgroups of CRGN and VRE carriers, compared to non-colonized patients (Table 1).

**Table 1:** Comparison of baseline characteristics between MDRO carriers and non-carriers.

	**Non carriers (*n* = 397)**	**CRGN carriers (*n* = 40)**	***p*-value**	**VRE carriers (*n* = 64)**	***p*-value**
Age (years); median (IQR)	68 (27)	72 (24.5)	0.341	72.5 (20)	0.120
Age ≥65 years; *n* (%)	220 (55.4)	27 (67.5)	0.142	45 (70.3)	**0.025**
Males; *n* (%)	214 (53.9)	19 (47.5)	0.439	35 (54.7)	0.907
LOS before rectal swabbing (days); median (IQR)	6 (8)	12.5 (20)	** <0.001**	10 (16.5)	** <0.001**
Ward type; *n* (%)					
•Internal medicine	230 (57.9)	19 (47.5)	0.204	44 (68.8)	0.102
•Surgery	167 (42.1)	21 (52.5)		20 (31.3)	
•Transfer from other hospital; *n* (%)	23 (10.1)	3 (27.3)	0.075	5 (17.9)	0.217
Indwelling medical devices; *n* (%)					
•Central line	16 (7.1)	1 (9.1)	0.808	4 (13.8)	0.212
•Urine catheter	75 (33.2)	7 (63.6)	**0.038**	9 (31.0)	0.816
•Pacemaker	8 (3.6)	2 (18.2)	**0.019**	1 (3.5)	0.977
•Other prosthetic material	40 (17.7)	2 (18.2)	0.967	7 (24.1)	0.400
•Chronic immobilization; *n* (%)	14 (6.2)	2 (18.2)	0.122	1 (3.5)	0.554
Last year; *n* (%)					
•Hospitalization	109 (48.2)	10 (90.9)	**0.007**	14 (50.0)	0.930
•ICU admission	13 (5.8)	4 (36.4)	** <0.001**	0 (0.0)	0.192
•Chemotherapy	32 (14.2)	4 (36.4)	**0.045**	6 (20.7)	0.353
•Immunosuppressive therapy	22 (9.7)	2 (18.2)	0.364	4 (13.8)	0.497
•Antibiotic treatment	132 (58.7)	11 (100.0)	**0.006**	15 (53.6)	0.606
Comorbidities; n (%)					
•Chronic kidney disease	23 (10.2)	1 (9.1)	0.907	5 (17.9)	0.221
•Diabetes	57 (25.3)	4 (36.4)	0.415	11 (39.3)	0.116
•Dermatologic lesions	41 (18.1)	3 (27.3)	0.447	1 (3.5)	**0.045**
•Hematological malignancies	11 (4.9)	1 (9.1)	0.533	1 (3.5)	0.734
•Solid organ malignancy	34 (15.0)	4 (36.4)	0.060	8 (27.6)	0.086
•Metastatic disease	16 (7.1)	3 (27.3)	**0.016**	5 (17.2)	0.061
•Neurologic disease	35 (15.5)	0 (0.0)	0.157	3 (10.3)	0.464
•Heart failure	30 (13.3)	2 (18.2)	0.642	7 (25.0)	0.097
•Myocardial infarct	26 (11.5)	2 (18.8)	0.503	4 (14.3)	0.667
•Chronic liver disease	8 (3.5)	0 (0.0)	0.526	1 (3.5)	0.980
•Cerebrovascular disease	22 (9.8)	1 (9.1)	0.937	0 (0.0)	0.077
•Peripheral vascular disease	32 (14.2)	2 (18.2)	0.710	5 (17.3)	0.657
•Chronic obstructive pulmonary disease	30 (13.3)	3 (27.3)	0.193	4 (14.3)	0.889
•Modified CCI; median (IQR)	2 (4)	4 (8)	**0.049**	2 (6)	0.352
Outcomes					
•Resistant pathogen in blood after rectal swabbing; *n* (%)	8 (2.0)	4 (10.0)	**0.003**	5 (7.8)	**0.009**
•Resistant pathogen in clinical culture after rectal swabbing; *n* (%)	28 (7.1)	12 (30.0)	** <0.001**	7 (10.9)	0.276
•Hospital LOS (days); median (IQR)	12 (13)	25 (38.5)	** <0.001**	20.5 (21)	** <0.001**
•LOS after rectal swabbing (days); median (IQR)	5 (10)	7 (13.5)	0.089	5 (12)	0.436
Mortality; *n* (%)	19 (4.8)	16 (40.0)	** <0.001**	10 (15.9)	** <0.001**

*IQR, interquartile range; LOS, length of stay; ICU, intensive care unit; CCI, Charlson comorbidity index; CRGN, carbapenem-resistant Gram-negative bacteria; VRE, vancomycin-resistant enterococci*.

*Bold indicates significant differences (p ≤ 0.050) of ANOVA for continuous variables and x^2^ analysis for categorical variables*.

*Numbers may not correspond to the total due to missing numbers*.

*(n = 491)*.

Table 2 presents the results of the univariable analysis on the association between potential risk factors and CRGN and VRE carriage. The detection of VRE carriage was associated with age over 65 years (OR: 1.9 [95%CI: 1.1, 3.4]) and LOS before rectal swab (OR: 1.1 [95%CI: 1.0, 1.1]). In a multivariate logistic regression model, these variables were also independently associated with VRE carriage [adjusted OR: 2.4 [95%CI: 1.3, 4.5] and 1.1 [95%CI: 1.0, 1.1], respectively]. CRGN carriage was associated with LOS before rectal swab [OR: 1.1 (95%CI: 1.0, 1.1)], hospitalization [OR: 10.4 (95%CI: 1.3, 82.3)] and ICU admission during the past 1 year [OR: 9.3 (95%CI: 2.4, 36.0)], presence of a urinary catheter [OR: 3.5 (95%CI: 1.0, 12.4)] and pacemaker [OR: 6.0 (95%CI: 1.1, 32.6)], metastatic disease [OR: 4.9 (95%CI: 1.2, 20.4)], and high modified CCI [OR: 1.2 (95%CI: 1.0, 1.3)]. When these variables were included in the multivariable model, none of them were identified as an independently associated risk factor for CRGN carriage.

**Table 2:** Associations between potential risk factors and carriage of CRGN and VRE.

	**CRGN carriers**			**VRE carriers**		
	n^*^	**OR (95% CI)**	***p*-value**	n^*^	**Crude OR (95% CI)**	***p*-value**
Age ≥65 years	437	1.7 (0.8, 3.3)	0.145	**461**	**1.9 (1.1, 3.4)**	**0.027**
LOS before rectal swabbing	**437**	**1.1 (1.0, 1.1)**	** <0.001**	**461**	**1.1 (1.0, 1.1)**	** <0.001**
**Indwelling medical devices**						
•Urine catheter	**237**	**3.5 (1.0, 12.4)**	**0.050**	255	0.9 (0.1, 2.1)	0.817
•Pacemaker	**236**	**6.0 (1.1, 32.6)**	**0.037**	254	1.0 (0.1, 8.0)	0.977
**Last year**						
Hospitalization	**237**	**10.4 (1.3, 82.3)**	**0.027**	254	1.1 (0.5, 2.4)	0.930
•ICU admission	**236**	**9.3 (2.4, 36.0)**	**0.001**	240	NA	NA
•Chemotherapy	237	3.5 (1.0, 12.5)	0.058	255	1.6 (0.6, 4.2)	0.356
•Antibiotic treatment	143	NA	NA	253	0.8 (0.4, 1.8)	0.607
**Comorbidities**						
•Chronic kidney disease	237	0.9 (0.1, 7.2)	0.907	254	1.9 (0.7, 5.5)	0.228
•Diabetes	236	1.7 (0.5, 6.0)	0.419	253	1.9 (0.8, 4.3)	0.121
•Dermatologic lesions	237	1.7 (0.4, 6.7)	0.452	255	0.2 (0.0, 1.2)	0.077
•Hematological malignancies	237	2.0 (0.1, 16.7)	0.540	255	0.7 (0.1, 5.6)	0.735
•Solid organ malignancy	237	3.2 (0.9, 11.6)	0.073	255	2.2 (0.9, 5.3)	0.092
•Metastatic disease	**237**	**4.9 (1.2, 20.4)**	**0.028**	255	2.7 (0.9, 8.1)	0.070
•Neurologic disease	202	NA	NA	255	0.6 (0.2, 2.2)	0.468
•Heart failure	237	1.5 (0.3, 7.1)	0.644	254	2.2 (0.9, 5.6)	0.104
•Myocardial infarct	237	1.7 (0.4, 8.3)	0.508	254	1.3 (0.4, 4.0)	0.668
•Chronic liver disease	229	NA	NA	255	1.0 (0.1, 8.1)	0.980
•Cerebrovascular disease	235	0.9 (0.1, 7.5)	0.937	231	NA	NA
•Peripheral vascular disease	237	1.4 (0.3, 6.5)	0.711	255	1.3 (0.4, 3.6)	0.658
•Chronic obstructive pulmonary disease	236	2.4 (0.6, 9.7)	0.206	253	1.1 (0.4, 3.3)	0.889
Modified CCI	**237**	**1.2 (1.0, 1.3)**	**0.037**	255	1.1 (1.0, 1.2)	0.069

*OR, odds ratio; CI, confidence interval; LOS, length of stay; ICU, intensive care unit; CCI, Charlson comorbidity index; NA, not applicable. Bold indicates significant differences (p ≤ 0.050)*.

* *Numbers do not correspond to the total in every risk factor, due to missing data*.

Table 3 shows the multivariable analysis estimating the effect of CRGN and VRE rectal carriage on hospital outcomes, after adjusting for sex, age, and modified CCI. Carrying CRGN was associated with 38 days increased hospital LOS (β-coef: 38.0, [95%CI: 22.6, 53.4]) and 11 days increase in LOS after rectal swabbing (β-coef: 11.4, [95%CI: 1.6, 21.2]). More importantly, it is associated with a 3.5-fold increased risk for acquiring a resistant pathogen after rectal swabbing (RR: 3.5, [95%CI 1.2, 9.9]) and with a 6-fold increase risk for mortality (RR: 6.1, [95%CI: 2.1, 17.9]). We then separately examined different CRGN subgroups in order to identify the specific pathogen underlying the observed associations; sample size was marginal for firm conclusions in some cases, but CR-enterobacteriaceae carriage was predictive of all outcomes (Supplementary Table 1). Regarding VRE carriers, although a statistical significant risk was found for hospital LOS, resistant pathogen in blood after rectal swabbing, and mortality in the crude model (RR: 9.9, [95%CI: 4.8, 15.1], RR: 3.9, [95%CI: 1.3, 11.5] and RR: 3.3, [95%CI: 1.6, 6.8] respectively), statistical significance was in all circumstances not shown after adjusting for confounders.

**Table 3:** Risk of CRGN and VRE carriage for hospital outcomes.

	**CRGN carriers**				**VRE carriers**			
	***n***	**Crude β-coef (95% CI)**	***n***	**Adjusted β-coef (95% CI)**	***n***	**Crude β-coef (95% CI)**	***n***	**Adjusted β-coef (95% CI)**
**HOSPITAL LOS (DAYS)**								
•Total	437	**25.0 (17.6, 32.4)**	237	**38.0 (22.6, 53.4)**	**437**	**9.9 (4.8, 15.1)**	**237**	4.7 (-3.2, 12.7)
•After rectal swabbing		**7.2 (2.4, 12.1)**		**11.4 (1.6, 21.2)**		1.3 (-2.3, 5.0)		0.4 (-6.1, 5.3)
	***n***	**Crude RR (95% CI)**	***n***	**Adjusted RR (95% CI)**	***n***	**Crude RR (95% CI)**	***n***	**Adjusted RR (95% CI)**
**RESISTANT PATHOGEN AFTER RECTAL SWABBING**								
•In blood	437	**5.0 (1.6, 15.8)**	237	NA	437	**3.9 (1.3, 11.5)**	237	1.7 (0.2, 15.1)
•In clinical culture		**4.3 (2.4, 7.7)**		**3.5 (1.2, 9.9)**		1.6 (0.7, 3.4)		0.9 (0.2, 3.7)
**Mortality**		**8.4 (4.7, 14.9)**		**6.1 (2.1, 17.9)**		**3.3 (1.6, 6.8)**		0.5 (0.1, 3.3)

*RR, relative risk; β-coef, beta coefficients; CI, confidence interval; LOS, length of stay; CR, carbapenem-resistant; VRE, vancomycin-resistant enterococci; NA, not applicable*.

*Models were adjusted for sex, age and modified Charlson comorbidity index. The values in bold are statistically significant*.

## Discussion

In the present study, we calculated simultaneously, for the first time, prevalence rates for VRE and CRGN carriage among inpatients of a Greek tertiary hospital and recognized prolonged hospitalization and age as independent risk factors for VRE carriage. We also showed that CRGN carriage is associated with increased risk of acquiring a resistant pathogen after rectal swabbing, prolonged hospital stays, and increased mortality.

The present study revealed a high prevalence of VRE and CRGN carriage among inpatients of our hospital (13.0 and 8.2% respectively). A previous study from the same hospital had determined a VRE carriage rate of 14.3% (29). Other studies have reported carriage rates that varied with geographic location and the general condition of patients (critically ill or not) (16, 17, 30–33). Greece is regularly regarded as an environment with high-selection pressure for the emergence of extensively drug-resistant Gram-negative bacilli in Europe, due to the over-consumption of antimicrobials both in the community and in the hospitals (34).

The multivariable analysis of potential risk factors showed that prolonged hospitalization and advanced age represent independent risk factors for VRE carriage. VRE can survive on environmental surfaces for a long time, and environmental contamination has been identified as a potential risk factor for VRE transmission to healthcare workers' hands and gloves and, subsequently, to patients (35, 36). Hand hygiene compliance rates in Greek hospitals have been reported to range from 22 to 43% (37–39). Since Greece is currently in the midst of a financial crisis, all Greek hospitals suffer from reductions in nursing and cleaning personnel, which may lead to compromises in infection control practices (40–42). All these factors might play a role in the further spread of VRE, especially in cases of prolonged hospitalization. In addition, long hospital stay may be associated with increased antibiotic consumption, which can also contribute to VRE selection and carriage (43). Moreover, the association of old age with VRE carriage may be attributed to several factors, such as alterations of the immune system, malnutrition, and social and economic factors (32, 44–46).

The univariate analysis revealed several risk factors for CRGN carriage: presence of indwelling devices (urinary catheter, pacemaker), CCI, metastatic disease, prolonged hospitalization before the fecal swab sampling, last year hospitalization, and ICU admission. Although none of these factors were independently associated with CRGN carriage in the multivariable logistic regression model, they all deserve to be considered. Indwelling devices are related to disease severity (47) and are recognized risk factors for healthcare-associated carriage and infection with MDR pathogens (48). Furthermore, serious underlying disease as a risk factor for CRGN carriage has been previously described (49) and can be explained by the patients' exposure to invasive procedures, as well as from impaired host defenses and extensive use of antibiotics. Almost invariably, these patients have longer hospitalization than patients with less severe illnesses (4). Also, previous hospitalization was identified as a risk factor for CRGN carriage. Our hospital is a referral center, where patients from all over the country are admitted for treatment. The majority of these patients have complex medical diseases, prolonged exposure to a healthcare setting, and an extensive use of antibiotics.

Gut carriage of CRGN was independently associated with increased hospital LOS, risk of acquiring a resistant pathogen after rectal swabbing, and mortality. Several studies have demonstrated that gut carriage and subsequent colonization by *K. pneumoniae* in hospitalized patients is associated with a greater risk of infection by the colonizing strains (50, 51). Little is known, though, about the mechanisms that promote progression from carriage to infection. Increased total LOS among CRE carriers has been previously described (19, 49), as well as the association with increased rate of CRE infections and high mortality (19, 20). Previous research has demonstrated that CRE infections are associated more often with sepsis and increased early mortality rate (52), particularly in vulnerable patients such as pediatric, geriatric, immunosuppressed, hospitalized, and chronically ill (53, 54).

Our study has some limitations. We might have underestimated the prevalence of OXA-48 producers that weakly hydrolyze carbapenems (MIC for meropenem lower than 1 mg/L) because of the selective media we used for the culture (55). Moreover, our study lacks enough power to establish a causal relationship between possible risk factors and MDRO carriage, since the study design was cross-sectional. We were also not able to gather detailed information regarding previous exposure to antibiotics such as carbapenems and vancomycin or to assess their impact on MDRO carriage. In addition, we didn't collect subsequent rectal samples in order to differentiate between transient carriage and colonization. More importantly, we did not collect the required data in order to define specific types of infections from resistant pathogens; instead, we collected data on isolation of a subsequent resistant pathogen from a clinical sample. Our analysis was limited to all-cause, rather than attributable, mortality. Thus, we are unable to determine whether the mortality rates of CRGN carriers were directly attributable to infection or were more likely to occur in patients with other fatal illnesses. Furthermore, although statistically significant conclusions were produced, confidence intervals were wide due to the relatively small sample size. Finally, although we tried to incorporate all known potential risk factors for carriage, infection, and mortality, we acknowledge that residual confounding from unmeasured covariates is still possible.

Nevertheless, the results of this study provide valuable information about the CRGN and VRE carriage burden in our hospital and can be used for improving our infection control strategy. After this study, the infection control team has been reinforced with more personnel. The health care employees of our hospital have been informed about the findings of the study and further educational activities on effective infection control practices have been provided. A new point prevalence study of MDRO carriage is scheduled for the first trimester of 2020 in order to compare results and assess the impact of the intensified infection control effort.

In conclusion, VRE and CRGN represent a serious public health problem and carrier patients represent a silent threat for hospitals. Efforts to limit the spread of MDRO need to be optimized.

## Data Availability Statement

The datasets generated for this study are available on request to the corresponding author.

## Ethics Statement

The studies involving human participants were reviewed and approved by Institutional Review Board, University General Hospital Attikon. The patients/participants provided their written informed consent to participate in this study.

## Author Contributions

SP, AA, and SV contributed to the study design and reviewed the manuscript. AV and PK contributed to the data analysis and manuscript preparation. SV and AT contributed to the data analysis. PV and MK collected and reviewed patients' data and contributed to the data analysis.

### Conflict of Interest

The authors declare that the research was conducted in the absence of any commercial or financial relationships that could be construed as a potential conflict of interest.

## References

[1] Solomon SL Oliver KB. Antibiotic resistance threats in the United States: stepping back from the brink. Am Fam Physician. (2014) 89:938–41. 25162160

[2] Sheng WH Chie WC Chen YC Hung CC Wang JTCS. Impact of nosocomial infections on medical costs, hospital stay, and outcome in hospitalized patients. J Formos Med Assoc. (2005) 104:318–26. 15959598

[3] Cassini A Högberg LD Plachouras D Quattrocchi A Hoxha A Simonsen GS. Attributable deaths and disability-adjusted life-years caused by infections with antibiotic-resistant bacteria in the EU and the European Economic Area in 2015: a population-level modelling analysis. Lancet Infect Dis. (2019) 19:56–66. 10.1016/S1473-3099(18)30708-4 30409683 PMC6300481

[4] Safdar N Dennis G Maki. Review the commonality of risk factors for nosocomial colonization and infection with antimicrobial-resistant Staphylococcus aureus, enterococcus, gram-negative bacilli, Clostridium difficile, and Candida. Ann Intern Med. (2002) 136:834–44. 10.7326/0003-4819-136-11-200206040-00013 12044132

[5] Akova M Daikos GL Tzouvelekis L Carmeli Y. Interventional strategies and current clinical experience with carbapenemase-producing Gram-negative bacteria. Clin Microbiol Infect. (2012) 18:439–48. 10.1111/j.1469-0691.2012.03823.x 22507111

[6] ECDC Annual Epidemiological Report: Antimicrobial Resistance and Healthcare-Associated Infections 2014. Stocholm: ECDC (2015).

[7] Kontopoulou K Iosifidis E Antoniadou E Tasioudis P Petinaki E Malli E. The clinical significance of carbapenem-resistant *Klebsiella pneumoniae* rectal colonization in critically ill patients: from colonization to bloodstream infection. J Med Microbiol. (2019) 68:326–35. 10.1099/jmm.0.000921 30688629

[8] Papadimitriou-Olivgeris M Christofidou M Fligou F Bartzavali C Vrettos T Filos KS. The role of colonization pressure in the dissemination of colistin or tigecycline resistant KPC-producing *Klebsiella pneumoniae* in critically ill patients. Infection. (2014) 42:883–90. 10.1007/s15010-014-0653-x 25008195

[9] Papadimitriou-Olivgeris M Drougka E Fligou F Kolonitsiou F Liakopoulos A Dodou V. Risk factors for enterococcal infection and colonization by vancomycin-resistant enterococci in critically ill patients. Infection. (2014) 42:1013–22. 10.1007/s15010-014-0678-1 25143193

[10] Metallidis S Chatzidimitriou M Tsona A Bisiklis A Lazaraki G Koumentaki E. Vancomycin-resistant enterococci, colonizing the intestinal tract of patients in a university hospital in Greece. Brazilian J Infect Dis. (2006) 10:179–84. 10.1590/S1413-86702006000300005 17568849

[11] Kofteridis DP Valachis A Dimopoulou D Maraki S Christidou A Mantadakis E. Risk factors for carbapenem-resistant *Klebsiella pneumoniae* infection/colonization: a case-case-control study. J Infect Chemother. (2014) 20:293–7. 10.1016/j.jiac.2013.11.007 24703709

[12] Sakka V Tsiodras S Galani L Antoniadou A Souli M Galani I. Risk-factors and predictors of mortality in patients colonised with vancomycin-resistant enterococci. Clin Microbiol Infect. (2008) 14:14–21. 10.1111/j.1469-0691.2007.01840.x 18005178

[13] Endtz HP Van Den Braak N Van Belkum A Kluytmans JAJW Koeleman JGM Spanjaard L. Fecal carriage of vancomycin-resistant enterococci in hospitalized patients and those living in the community in the Netherlands. J Clin Microbiol. (1997) 35:3026–31. 10.1128/JCM.35.12.3026-3031.1997 9399488 PMC230116

[14] Tokars JI1 Satake S Rimland D Carson L Miller ER Killum E. The prevalence of colonization with vancomycin-resistant Enterococcus at a Veterans' Affairs institution. Infect Control Hosp Epidemiol. (1999) 20:171–5. 10.1086/501606 10100542

[15] Gambarotto K Ploy MC Turlure P Grélaud C Martin C Bordessoule D. Prevalence of vancomycin-resistant enterococci in fecal samples from hospitalized patients and nonhospitalized controls in a cattle-rearing area of France. J Clin Microbiol. (2000) 38:620–4. 10.1128/JCM.38.2.620-624.2000 10655356 PMC86160

[16] Vidal-Navarro L Pfeiffer C Bouziges N Sotto A Lavigne JP. Faecal carriage of multidrug-resistant Gram-negative bacilli during a non-outbreak situation in a French university hospital. J Antimicrob Chemother. (2010) 65:2455–8. 10.1093/jac/dkq333 20813808

[17] Wiener-Well Y Rudensky B Yinnon AM Kopuit P Schlesinger Y Broide E. Carriage rate of carbapenem-resistant *Klebsiella pneumoniae* in hospitalised patients during a national outbreak. J Hosp Infect. (2010) 74:344–9. 10.1016/j.jhin.2009.07.022 19783067

[18] Tran DM Larsson M Olson L Hoang NTB Le NK Khu DTK. High prevalence of colonisation with carbapenem-resistant Enterobacteriaceae among patients admitted to Vietnamese hospitals: risk factors and burden of disease. J Infect [Internet]. (2019) 79:115–22. 10.1016/j.jinf.2019.05.013 31125639

[19] Tischendorf J De Avila RA Safdar N. Risk of infection following colonization with carbapenem-resistant Enterobactericeae: a systematic review. Am J Infect Control. (2016) 44:539–43. 10.1016/j.ajic.2015.12.005 26899297 PMC5262497

[20] McConville TH Sullivan SB Gomez-Simmonds A Whittier S Uhlemann AC. Carbapenem-resistant Enterobacteriaceae colonization (CRE) and subsequent risk of infection and 90-day mortality in critically ill patients, an observational study. PLoS ONE. (2017) 12:1–14. 10.1371/journal.pone.0186195 29023567 PMC5638409

[21] Pournaras S Poulou A Tsakris A. Inhibitor-based methods for the detection of KPC carbapenemase- producing Enterobacteriaceae in clinical practice by using boronic acid compounds. J Antimicrob Chemother. (2010) 65:1319–21. 10.1093/jac/dkq124 20395214

[22] European Committee on Antimicrobial Susceptibility Testing EUCAST EUCAST Guidelines for Detection of Resistance Mechanisms and Specific Resistances of Clinical and/or Epidemiological Importance Version 2.0. Basel.

[23] Giakkoupi P Pappa O Polemis M Vatopoulos AC Miriagou V Zioga A. Emerging *Klebsiella pneumoniae* isolates coproducing KPC-2 and VIM-1 carbapenemases. Antimicrob Agents Chemother. (2009) 53:4048–50. 10.1128/AAC.00690-09 19581459 PMC2737892

[24] Tacconelli E Cataldo MA Dancer SJ Angelis GDe Falcone M Frank U. ESCMID guidelines for the management of the infection control measures to reduce transmission of multidrug-resistant Gram-negative bacteria in hospitalized patients. Clin Microbiol Infect. (2013) 20:1–55. 10.1111/1469-0691.12427 24329732

[25] Morris-downes M Smyth EG Moore J Thomas T Fitzpatrick F Walsh J. Surveillance and endemic vancomycin-resistant enterococci : some success in control is possible. J Hosp Infect. (2010) 75:228–33. 10.1016/j.jhin.2010.01.004 20363048

[26] Charlson ME Pompei P Ales KLMC. A new method of classifying prognostic comorbidity in longitudinal studies: development and validation. J Chronic Dis. (1987) 40:373–83. 10.1016/0021-9681(87)90171-8 3558716

[27] Laksmi PW Harimurti K Setiati S Soejono CH Aries W Roosheroe AG. Management of immobilization and its complication for elderly. Acta Med Indones. (2008) 40:233–40. 19151453

[28] Anderson CL CN Principles of geriatric medicine and gerontology. In: Hazzard WR Blass JP Etinger HW Halter JB editor. New York, NY: McGraw-Hill (1999). p. 1565–75.

[29] Souli M Sakka V Galani I Antoniadou A Galani L Siafakas N. Colonisation with vancomycin- and linezolid-resistant Enterococcus faecium in a university hospital: molecular epidemiology and risk factor analysis. Int J Antimicrob Agents. (2009) 33:137–42. 10.1016/j.ijantimicag.2008.08.017 19013056

[30] Torres-Gonzalez P Cervera-Hernandez ME Niembro-Ortega MD Leal-Vega F Cruz-Hervert LP García-García L. Factors associated to prevalence and incidence of carbapenem-resistant enterobacteriaceae fecal carriage: a cohort study in a Mexican tertiary care hospital. PLoS ONE. (2015) 10:1–13. 10.1371/journal.pone.0139883 26431402 PMC4592225

[31] Swaminathan M Sharma S Blash SP Patel G Banach DB Phillips M. Prevalence and risk factors for acquisition of carbapenem-resistant enterobacteriaceae in the setting of endemicity. Infect Control Hosp Epidemiol. (2013) 34:809–17. 10.1086/671270 23838221

[32] Kim HS Kim DH Yoon HJ Lee WJ Woo SH Choi SP. Factors associated with vancomycin-resistant enterococcus colonization in patients transferred to emergency departments in Korea. J Korean Med Sci. (2018) 33:1–7. 10.3346/jkms.2018.33.e295 30473648 PMC6249167

[33] Karki S Houston L Land G Bass P Kehoe R Borrell S. Prevalence and risk factors for VRE colonisation in a tertiary hospital in Melbourne, Australia: a cross sectional study. Antimicrob Resist Infect Control. (2012) 1:31. 10.1186/2047-2994-1-31 23039285 PMC3523023

[34] Souli M Galani I Giamarellou H. Emergence of extensively drug-resistant pandrug-resistant Gram-negative bacilli. Eurosurveillance. (2008) 13:1–11. 19021957

[35] Duckro AN Blom DW Lyle EA Weinstein RA Hayden MK. Transfer of vancomycin-resistant enterococci via health care worker hands. Arch Intern Med. (2005) 165:302–7. 10.1001/archinte.165.3.302 15710793

[36] Boyce JM. Environmental contamination makes an important contribution to hospital infection. J Hosp Infect. (2007) 65 (Suppl. 2):50–4. 10.1016/S0195-6701(07)60015-2 17540242

[37] Astrinaki E Messaritaki A Mourtou E Niakas D Hand hygiene compliance in a Greek university hospital. Arch Hell Med. (2016) 33:639–44.

[38] Poulou A Voulgari E Vrioni G Xidopoulos G Pliagkos A Chatzipantazi V. Imported *Klebsiella pneumoniae* carbapenemase-producing *K. pneumoniae* clones in a Greek hospital: impact of infection control measures for restraining their dissemination. J Clin Microbiol. (2012) 50:2618–23. 10.1128/JCM.00459-12 22649010 PMC3421501

[39] Kouni S Kourlaba G Mougkou K Maroudi S Chavela B Nteli C. Assessment of Hand hygiene resources and practices at the 2 children's hospitals in Greece. Pediatr Infect Dis J. (2014) 33:e247–51. 10.1097/INF.0000000000000376 25361195

[40] Hugonnet S Harbarth S Sax H Duncan RA Pittet D. Nursing resources: a major determinant of nosocomial infection? Curr Opin Infect Dis. (2004) 17:329–33. 10.1097/01.qco.0000136931.83167.d2 15241077

[41] Economou C Kaitelidou D Kentikelenis A Sissouras A Maresso A The Impact of the Financial Crisis on the Health System and Health in Greece. Copenhagen: WHO; European Observatory on Health Systems and Policies (2014). Available online at: http://www.euro.who.int/__data/assets/pdf_file/0007/266380/The-impact-of-the-financial-crisis-on-the-health-system-and-health-in-Greece.pdf (accessed March 8, 2020).

[42] Kousouli E Zarkotou O Politi L Polimeri K Vrioni G Themeli-Digalaki K. Infection control interventions affected by resource shortages: impact on the incidence of bacteremias caused by carbapenem-resistant pathogens. Eur J Clin Microbiol Infect Dis. (2018) 37:43–50. 10.1007/s10096-017-3098-1 28879405

[43] McKinnell JA Kunz DF Moser SA Vangala S Tseng CH Shapiro M. Patient-level analysis of incident vancomycin-resistant enterococci colonization and antibiotic days of therapy. Epidemiol Infect. (2016) 144:1748–55. 10.1017/S0950268815003118 27125574 PMC5943038

[44] Tacconelli E De Angelis G Cataldo MA Mantengoli E Spanu T Pan A. Antibiotic usage and risk of colonization and infection with antibiotic-resistant bacteria: a hospital population-based study. Antimicrob Agents Chemother. (2009) 53:4264–9. 10.1128/AAC.00431-09 19667289 PMC2764223

[45] High KP. Infection as a cause of age-related morbidity and mortality. Ageing Res Rev. (2004) 3:1–14. 10.1016/j.arr.2003.08.001 15163100

[46] McEvoy SP Plant AJ Pearman JW. Risk factors for the acquisition of vancomycin-resistant enterococci during a single-strain outbreak at a major Australian teaching hospital. J Hosp Infect. (2006) 62:256–8. 10.1016/j.jhin.2005.06. 16257091

[47] Zhao ZC Xu XH Liu MB Wu J Lin J Li B. Fecal carriage of carbapenem-resistant Enterobacteriaceae in a Chinese university hospital. Am J Infect Control. (2014) 42:e61–4. 10.1016/j.ajic.2014.01.024 24773806

[48] Rosenthal VD Maki DG Salomao R Álvarez-Moreno C Mehta Y Higuera F. Device-associated nosocomial infections in 55 intensive care units of 8 developing countries. Ann Intern Med. (2006) 145:582–91. 10.7326/0003-4819-145-8-200610170-00007 17043340

[49] Asai N Sakanashi D Suematsu H Kato H Hagihara M Nishiyama N. The epidemiology and risk factor of carbapenem-resistant enterobacteriaceae colonization and infections: case control study in a single institute in Japan. J Infect Chemother. (2018) 24:505–9. 10.1016/j.jiac.2018.02.005 29548627

[50] Martin RM Cao J Brisse S Passet V Wu W Zhao L. Molecular epidemiology of colonizing and infecting isolates of *Klebsiella pneumoniae*. mSphere. (2016) 1:1–12. 10.1128/mSphere.00261-16 27777984 PMC5071533

[51] Gorrie CL Mirceta M Wick RR Edwards DJ Nicholas R Strugnell RA. Gastrointestinal carriage is a major reservoir of klebsiella pneumoniae infection in intensive care patients. Clin Infect Dis. (2017) 65:208–15. 10.1093/cid/cix270 28369261 PMC5850561

[52] Tumbarello M Viale P Viscoli C Trecarichi EM Tumietto F Marchese A. Predictors of mortality in bloodstream infections caused by *Klebsiella pneumoniae* carbapenemase-producing K. pneumoniae: Importance of combination therapy. Clin Infect Dis. (2012) 55:943–50. 10.1093/cid/cis588 22752516

[53] Kalpoe JS Sonnenberg E Factor SH del Rio Martin J Schiano T Patel G. Mortality associated with carbapenem-resistant *Klebsiella pneumoniae* infections in liver transplant recipients. Liver Transplant. (2012) 18:468–74. 10.1002/lt.23374 22467548

[54] Wang Z Qin RR Huang L Sun LY. Risk factors for carbapenem-resistant *Klebsiella pneumoniae* infection and mortality of *Klebsiella pneumoniae* infection. Chin Med J. (2018) 131:56–62. 10.4103/0366-6999.221267 29271381 PMC5754959

[55] Bakthavatchalam YD Anandan S VB. Laboratory detection and clinical implication of oxacillinase-48 like carbapenemase: the hidden threat. J Glob Infect Dis. (2016) 8:41–50. 10.4103/0974-777X.176149 27013843 PMC4785756

