# Supplementary material for: Gastrointestinal Carriage of Vancomycin-Resistant Enterococci and Carbapenem-Resistant Gram-Negative Bacteria in an Endemic Setting: Prevalence, Risk Factors, and Outcomes
Source: Front Public Health. 2020 Mar 18;8:55. doi: 10.3389/fpubh.2020.00055 (PMC7093565; doi:10.3389/fpubh.2020.00055)
Supplement: Supplementary file 1 [file Table_1.pdf]

Supplementary Material

Supplementary Table 1: Risk of CRGN subgroups carriage on hospital outcomes.

	**Hospital LOS (days)**				**Resistant pathogen after rectal swabbing**				**Mortality**	
	**Total**		**After rectal swabbing**		**In blood**		**In clinical culture**			
	Crude β-coef (95% CI)	Adjusted β-coef (95% CI)	Crude β-coef (95% CI)	Adjusted β-coef (95% CI)	Crude RR (95% CI)	Adjusted RR (95% CI)	Crude RR (95% CI)	Adjusted RR (95% CI)	Crude RR (95% CI)	Adjusted RR (95% CI)
	N=437	N=237	N=437	N=237	N=437	N=237	N=437	N=237	N=437	N=237
CR- enterobacteriaceae (n=29)	**31.6 (23.0, 40.2)**	**40.2 (24.1, 56.2)**	**10.0 (4.3, 15.6)**	**11.1 (0.9, 21.3)**	**5.1 (1.4, 18.3)**	NA	**4.4 (2.2, 8.4)**	**3.8 (1.3, 10.5)**	**8.6 (4.7, 16.0)**	**5.7 (1.8, 18.1)**
CR-P. aeruginosa (n=6)	7.6 (-7.5, 22.7)	15.8 (-25.2, 56.9)	0.3 (-10.7, 11.4)	14.9 (-15.2, 44.9)	NA	NA	2.4 (0.4, 14.7)	NA	**7.0 (2.1, 23.4)**	**9.8 (4.1, 23.6)**
CR-A. baumannii (n=9)	5.2 (-7.1, 17.6)	8.6 (-20.3, 37.5)	0.3 (-8.7, 9.3)	5.4 (-15.7, 26.6)	5.5 (0.8, 39.6)	NA	**4.7 (1.8, 12.7)**	**5.1 (1.0, 24.6)**	**7.0 (2.5, 19.4)**	**8.1 (1.4, 48.0)**

RR: relative risk; β-coef: beta coefficients; CI: confidence interval; LOS: length of stay; CR: carbapenem-resistant; VRE: vancomycin-resistant enterococci; NA: not applicable
All models were adjusted for sex, age and modified Charlson comorbidity index.
